# A determination of patient preferences for China online outpatient follow-up clinics by using discrete choice experiment: an exploratory study

**DOI:** 10.3389/fpubh.2025.1508369

**Published:** 2025-03-24

**Authors:** Nan Chen, Dan Bai, Jing Ning

**Affiliations:** School of Management, Shanghai University, Shanghai, China

**Keywords:** telehealth, internet hospital, online outpatient follow-up clinic, stated preferences, discrete choice experiment

## Abstract

**Objective:**

Internet hospitals as a telehealth platform are rapidly growing in China both in quality and scale. This study aims to explore Chinese patients’ preferences, trade-offs and willingness to pay for attributes of online outpatient follow-up clinics.

**Methods:**

A discrete choice experiment was designed to elicit respondents’ stated preferences for six attributes. The online survey was conducted between November 2023 and December 2023, and mixed logit models were used to analyze the data.

**Results:**

A total of 337 valid Chinese respondents were recruited. The results showed that an online outpatient follow-up clinic preferred by respondents could be described as low cost, provided by a tertiary Internet hospital, available same-day appointment, appointment with their own first diagnosing doctor, video appointment consultation, and payment by medical insurance. People with higher e-health literacy were more likely to accept an online outpatient follow-up appointment.

**Conclusion:**

Chinese respondents place a high value on waiting time for appointments, online payment methods, and continuity of online doctors. They were willing to pay more to avoid waiting a week for an online appointment. Our study provides valuable information for the telehealth policy-making and operation of Internet hospitals for healthcare sustainability.

## 1 Introduction

In recent years, China has witnessed the emergence and growing trend of Internet hospitals, which leverage telecommunication technology to provide healthcare services with the Internet access rate reaching 77.5% by December 2023 (1). According to the National Health Commission 2022, China had only 2.9 physicians and 3.5 nurses per 1,000 population, below the WHO’s recommended 4.45 health workers per 1,000. The hospital system in China is divided into three levels. Tertiary hospitals provide high-level specialized services and undertake high-level teaching research tasks. Secondary hospitals provide comprehensive medical services to multiple communities. Primary hospitals directly provide preventive, medical, healthcare, and rehabilitation services to communities. According to the National Health Statistics Yearbook 2022, 80% of Chinese tertiary hospitals were concentrated in urban areas. Therefore, China faces limited healthcare resources and geographic inequities in the allocation of healthcare resources.

Internet hospital as a telehealth platform integrates online and offline access, extends medical resources from traditional hospitals to the Internet and provides diverse telehealth services directly to patients (2–5). Due to specific policy restrictions, Internet hospitals mainly focus on providing domestic online outpatient follow-up clinics for individuals with common or chronic diseases, instead of initial consultations (6). Nevertheless, the development of Internet hospitals in China has strengthened the healthcare system by addressing gaps in outpatient services for remote region and relieving overcrowding in traditional hospitals (1, 4, 7). According to the National Health Statistics Yearbook 2023, China’s tertiary hospitals averaged 95% bed occupancy rates, with peak outpatient wait times exceeding 4 h. Furthermore, Internet hospitals improve the efficiency of patient care by providing convenient services such as online registration, access to electronic medical records, and online payment (3). In the context of infectious public health emergencies, Internet hospitals play a crucial role in efficiently screening potential patients, thereby safeguarding patients, healthcare personnel, and communities from infection risks (8).

While the Chinese people have been somewhat motivated by the COVID-19 pandemic to use Internet hospitals for health management (8–11), the overall use of Internet hospitals has been low. A post-pandemic survey carried out in China, indicated that 63.28% of the patients were familiar with using Internet hospitals to make outpatient appointments, but only 28.55% of them chose to use this service (12). Correspondingly, the effective utilization of online outpatient follow-up clinics provided by Internet hospitals is also low. Ma et al.’s (13) survey showed that 74.0% of Chinese healthcare professionals used telemedicine once a week, and the average duration of their participation in telemedicine services mainly lasted 11–30 min. The further development of Internet hospitals is limited by some practical factors. First, many Internet hospitals face challenges due to a shortage of online medical practitioners and insufficient health insurance coverage. Second, Internet hospital services have yet to reach the entire population due to uneven public acceptance. Patients who are not familiar with the Internet and mobile devices have difficulty using Internet hospital platforms. Third, the number of patients in Internet hospitals operated by offline hospitals has not increased rapidly, constrained by the influence of the hospitals themselves and patients’ preference for the traditional mode of medical care (2, 6). Therefore, to improve the quality of online outpatient follow-up clinics and better match patients’ demands, it is necessary to investigate what kind of online outpatient follow-up clinics Chinese patients prefer and what their priorities are when choosing this service.

Addressing patient preferences in healthcare service provision is a crucial aspect of health policy-making (14, 15). Several studies have explored patient preferences for different telehealth services using a stated preference method known as a discrete choice experiment (DCE) and other direct surveys (16–18). Discrete choice experiments (DCE) are a quantitative method used to elicit individuals’ preferences by presenting them with hypothetical scenarios involving different attributes and levels. Respondents are asked to choose their preferred option, allowing researchers to analyze trade-offs and estimate willingness to pay (WTP). These studies primarily investigated stakeholder groups’ perceptions of factors related to telehealth, such as cost, type of service platform, continuity of providers, response time, and clinicians’ attitudes. People’s perceived utility of the same factor differs across studies. For example, cost was identified as the most crucial attribute of the telemedicine service for diabetes management (16), while least in older Australians’ choice of telehealth services (17). Heterogeneity in preferences for telehealth has also been found in the literature, with individual characteristics (e.g., gender, age, income, experience) having an impact on people’s preferences and choices (16, 17, 19–24). For instance, according to Khairat et al. (20), patients from urban areas and males demonstrated a higher likelihood of choosing telemedicine over mHealth. In China, existing studies primarily focus on outlining trends and economic benefits of Internet hospitals, and there is limited information regarding patients’ specific preferences for online outpatient follow-up clinics. Given the distinctions in online outpatient follow-up clinics between China and other developed countries such as the UK and the US, coupled with differences in service costs, appointment difficulties, family doctor contracts (an agreement between a family or individual and a general practitioner or a team of healthcare providers, which offers primary healthcare services including preventive care, diagnosis, treatment, and health management in exchange for a certain fee or covered by insurance), reimbursement rates, and cultural factors, the crucial attributes that Chinese patients consider important remain unclear. This study represents an initial attempt to fill this gap, exploring Chinese people’s preferences and willingness to pay (WTP) for attributes of online outpatient follow-up clinics, evaluating the relative importance of attributes, and determining the impact of individual characteristics on the strength of preferences for these attributes. Besides, this study specifically focuses on domestic telehealth services within China’s internet hospital framework, excluding cross-border healthcare applications. However, the research results can be generalized in other similar healthcare environments and the research methodology is also instructive to different healthcare systems around the world.

The contribution of this study mainly lies in the following three aspects. First, to our knowledge, this is the first application of the DCE approach to investigate respondents’ preferences and WTP for online outpatient follow-up clinics in China. Second, two attributes (type of Internet hospital and continuity of online doctors) that have received little attention in the existing literature on preferences for telehealth in China are included in our DCE. Finally, this study extends respondents’ socio-demographic characteristics with their healthcare technology self-efficacy and e-health literacy.

## 2 Methods

### 2.1 DCE methodology

In recent years, discrete choice experiments (DCEs) based on random utility theory (RUT) (25) have emerged as the most commonly applied stated preference method in healthcare (26). They are employed to explore trade-offs among attributes of goods or services, estimate the monetary value of non-market commodities (27), and evaluate the relative importance of aspects of decision making related to health interventions or healthcare services (28, 29). In a DCE, respondents are presented with a series of choice sets and asked to choose a preferred alternative in each choice set (30). Each choice set consists of two or more alternatives defined by a set of attributes that vary across a specified and reasonable range of levels (30, 31). The relative strength of preferences for improvements in particular attributes can be quantified by analysing the choices that have been made. Moreover, with the inclusion of a cost attribute, they can estimate the marginal WTP for these attributes (30, 32). Additionally, DCEs can predict the market share of new products or services and the probability of uptake of particular alternatives (33), providing valuable insights for product pricing and policymaking.

### 2.2 Development of attributes and levels

The attributes and attribute levels were identified through comprehensive review of the literature on telehealth/telemedicine utilization, satisfaction, and preferences, and qualitative interview with experts from Chinese public Internet hospitals. These experts were selected from Chinese public Internet hospitals, like Zhongshan Hospital, Fudan University and Ruijin Hospital, Shanghai Jiao Tong University School of Medicine, who have been actively involved in the planning, implementation, and management of telehealth services. Six attributes were identified, each with two or three levels based on the results of a pre-experiment:

1. Cost. It refers to the cost of an online outpatient follow-up clinic appointment. Different cost levels are assigned for the three types of Internet hospitals based on a statistical survey in China.
2. Type of Internet hospital. There are three levels of public hospitals, encompassing primary hospitals (community health centers), secondary hospitals, and tertiary hospitals, which are also the corresponding initiators of public Internet hospitals.
3. Type of online doctor. This attribute indicates the continuity of the doctor-patient relationship, specifically whether the doctor is the one who first diagnosed the patient in the hospital.
4. Waiting time for an appointment. It represents the number of days to wait for an available online outpatient follow-up clinic appointment.
5. Communication mode. This attribute refers to the methods of consulting a doctor provided by Internet hospitals.
6. Online payment method. It refers to either payment by medical insurance or payment without medical insurance.

The attributes and attribute levels are shown in Table 1.

**Table 1:** DCE attributes and attribute levels.

Attribute	Level	Variable
Cost (Chinese Yuan, CNY)	Primary Internet hospital: 10, 20, 30	Cost
	Secondary Internet hospital: 18, 30, 40	Cost
	Tertiary Internet hospital: 25, 50, 60	Cost
Type of internet hospital	Tertiary Internet hospital [Reference]	Hospital3
	Secondary Internet hospital	Hospital2
	Primary (Community) Internet hospital	Hospital1
Type of online doctor	First diagnosing doctor [Reference]	DoctorOwn
	Non-first diagnosing doctor at the patient’s first diagnosing hospital	DoctorNF
	Non-first diagnosing doctor at the patient’s non-first diagnosing hospital	DoctorNN
Waiting time for an appointment	Today [Reference]	AppointToday
	3 days	Appoint3days
	A week	Appoint7days
Communication mode	Video consultation [Reference]	Video
	Voice (telephone) consultation	Telephone
	Image-text consultation	Imagetext
Online payment method	Payment by medical insurance [Reference]	MeInsurance
	Payment without medical insurance	NonMeInsurance

### 2.3 DCE design

An unlabeled DCE was employed to encourage respondents to make choices based on trade-offs among all attributes, instead of paying more attention to the label itself (28, 34). A D-efficiency design was created in SAS 9.4 software and a total of 36 choice sets were developed. To reduce the cognitive burden on each respondent, the 36 choice sets were blocked into three versions, each with 12 choice sets, and each respondent was randomly assigned to one version. Besides, an unforced DCE was employed which includes unforced choice, to avoid forcing respondents to make a choice between several potentially unattractive appointment options (33, 35). We also included a fixed opt-out option in each choice set to improve the fidelity of choice tasks and reduce the risk of overestimating the importance of attributes (36). Therefore, each choice set contained three options, two unlabeled online outpatient follow-up clinic appointment alternatives (Appointment A and Appointment B) and an opt-out option, in which the opt-out option was described as “none of the alternatives,” representing that respondents did not want to choose either of the two online follow-up clinics.

### 2.4 Questionnaire design

The questionnaire consisted of three parts. The first part included four validated scales (37–40) to measure the impact of latent variables of interest on patient preferences:

1. risk attitude (RA), which refers to an individual’s willingness to engage in behaviors that may involve potential harm or uncertainty. Liu et al. (41) explored patients’ preferences and choice behaviors in scheduling medical appointments, and demonstrated that risk attitudes mediate the impact of gender on the perception of the speed and quality of medical services through a DCE experiment.
2. healthcare technology self-efficacy (HTSE), which represents an individual’s confidence in their ability to use healthcare-related technologies effectively. Rahman et al. (40) proposed the concept of health technology self-efficacy in the healthcare context and demonstrated that health technology self-efficacy has a positive impact on the attitude towards the use of health technologies.
3. e-health literacy (EHEAL), which refers to the ability to read, use computers, search for information, understand health information, and apply it (37).
4. online privacy concerns (OPC), which reflects the level of worry and concern that patients have regarding the privacy and security of their personal health information when using online healthcare services. Weinrich et al. (36) included the level of patients’ online privacy concerns in survey, which explored the factors influencing patients’ preferences for video consultations during the COVID-19. The DCE experiment results showed that fewer online privacy concerns led to a higher utility from video consultations compared to in-clinic consultations.

With reference to the above-mentioned literature, the study used these four attitudinal and psychological tools to explore the internal reasons for patients’ preferences in the context of online outpatient follow-up clinic appointment in Internet hospitals in China.

**Figure 1:**
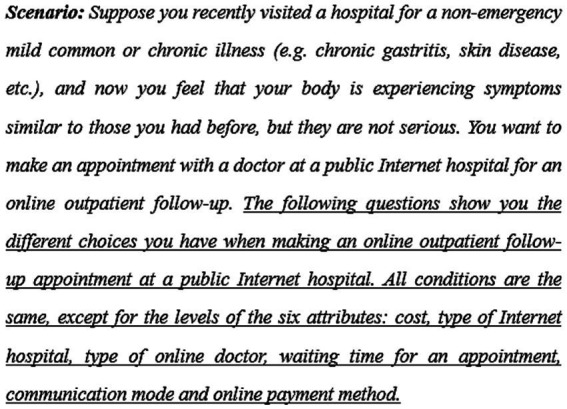
The scenario of DCE experiment. Participants are placed in the scenario described above to complete the DCE experiment. From the patients’ point of view, we analyze which attributes may have an impact on whether or not they choose an online follow-up appointment and what kind of online follow-up appointment they prefer.

**Figure 2:**
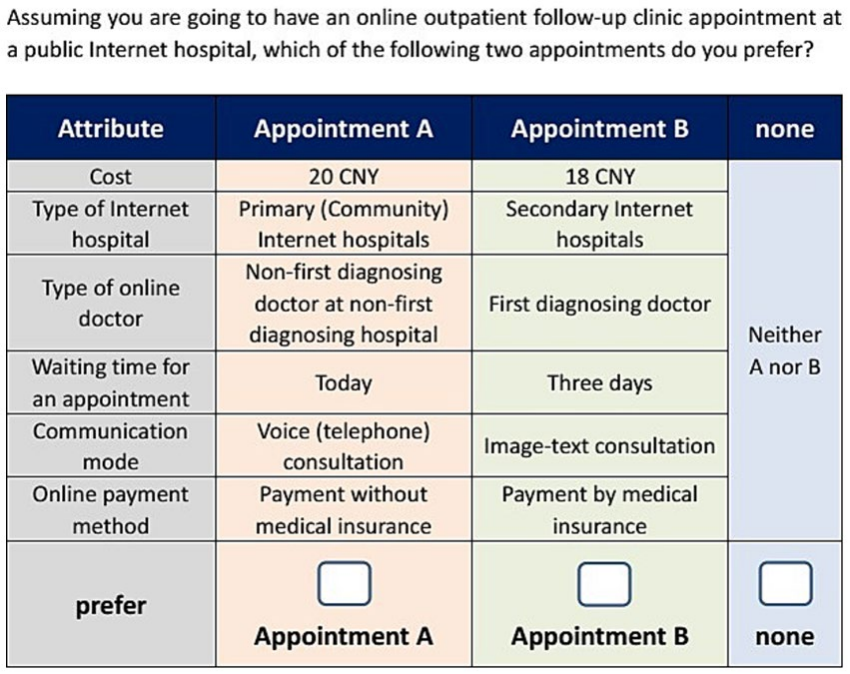
Example of choice set. There are two types of online outpatient follow-up clinic appointment for patients to choose. Patients can compare the two appointments horizontally in each of the following six dimensions: cost, type of Internet hospital, type of online doctor, waiting time for an appointment, communication mode, online payment method. If patients do not want to make an online follow-up clinic appointment, they can select none option.

In the second part, 13 DCE choice sets were presented, consisting of 12 formal choice sets and one repeated DCE choice set (used to test internal validity). Respondents were first introduced to the DCE choice scenarios, the meaning of the attributes and respective levels, and then asked to choose the preferred alternative in each choice set. The third part included 13 questions about basic demographic information, including gender, age and monthly income. The description of the DCE scenario and an example of a DCE choice set are shown in Figures 1, 2, respectively. Besides, this study was approved by the Ethics Committee of Shanghai University (Approval code: ECSHU 2023–109, 21 November 2023). The ethics statement document was included in the Supplementary materials.

### 2.5 Data collection

The survey was administered through Wenjuanxing,^1^ China’s largest professional survey platform with ISO 26362 certification. We inputted the designed questionnaire into this platform for publication, and the platform was responsible for the distribution and collection of the questionnaire based on our requirements. The platform randomly distributes the survey questionnaires to the online population. By sharing the questionnaire links in communities and inviting the online population to participate in the survey through message notifications, we recruited as many respondents as possible.

We define our target patients as Chinese adults (≥18 years) with potential needs for online outpatient follow-up services (have knowledge of or have ever used telehealth medical services) and the ability to access the Internet, regardless of prior telemedicine experience. This inclusive approach allows us to capture both current and prospective users’ preferences and make our research more generalizable. Meanwhile, the patients in our survey achieved a gender balance, which is consistent with the gender ratio in 2020 National Census.

The sample size was determined applying rules of thumb based on the number of attribute levels in Equation 1 (26, 42):


(1) $$N>\frac{500c}{t\times a\text{.}}$$

Where t denotes the number of choice tasks, a denotes the number of alternatives, and c the number of levels of attributes. In this DCE, c=3 (the maximum number of levels of an attribute is 3), t=12 (each version of the questionnaire contains 12 DCE choice sets, excluding one repeated DCE choice set used to test internal validity), and a=2 (each choice task contains two alternatives, excluding the opt-out option). Therefore, a minimum of 63 respondents is required for each version of the questionnaire. The online survey was formally conducted through an online platform from November 2023 to December 2023. The platform offered incentives to motivate sufficient respondents to participate online, which was a common approach used by researchers, like the survey conducted by Wong et al. (43) to improve response rate and the quality of questionnaires (44). We also took preventive measures to reduce the negative impacts of this incentive approach, such as giving prompts during the experiment, and eliminating invalid samples after the experiment. The platform was also commissioned to collect a sample covering as wide an age range as possible, and with a balanced gender ratio. Given the percentage of invalid responses and to analyse the heterogeneity of preferences, a total of 337 valid samples were collected, including 109 respondents for version A1, 114 respondents for version A2, and 114 respondents for version A3.

## 3 Statistical analysis

Mixed logit (MXL) models were used to analyze the respondents’ online stated preference data, which relaxes the assumption of Independence of Irrelevant Alternatives (IIA), accommodates the panel nature of the DCE data by allowing correlation of subjects who have made repeated choices, and captures the heterogeneity of preferences across individuals by allowing model coefficients to vary across respondents (45–47).

- *Main effects model:* In the main effects mixed logit model, the cost attribute is defined as a continuous variable, while the other attributes are considered categorical variables. This decision is based on the understanding that treating cost as a categorical variable does not improve the model (48). Data are coded using the dummy coding method, as recommended by Hu et al. (49). As shown in Equation 2, the observable utility $V_{ij}$that respondent i chooses the online outpatient follow-up appointment j can be described as a function of the attribute and attribute levels (excluding the reference levels of the categorical attributes):

(2) $$\begin{array}{l} V_{ij}=\beta_{1}\cos t_{ij}+\beta_{2}\mathrm{Hospital}2_{ij}+\beta_{3}\mathrm{Hospital}1_{ij}+\beta_{4}\mathrm{DoctorN}F_{ij}+\\ \beta_{5}\mathrm{DoctorN}N_{ij}+\beta_{6}\mathrm{Appoint}3\mathrm{day}s_{ij}+\beta_{7}\mathrm{Appoint}7\mathrm{day}s_{ij}+\\ \beta_{8}\mathrm{Telephon}e_{ij}+\beta_{9}\mathrm{Imagetex}t_{ij}+\beta_{10}\mathrm{NonMeInsuranc}e_{ij} \end{array}$$

The meaning of the variables is shown in Table 1.

- *Interaction model:* Interaction models capture the effect of individual characteristics on choice preferences. This effect can be estimated by including the interaction of individual characteristics with the attributes in the main effects mixed logit model. Taking gender as an example, the expected utility of including the gender interaction variable can be expressed as follows in Equation 3:

(3) $$\begin{array}{l} V_{ij}=\beta_{1}\cos t_{ij}+\beta_{2}\mathrm{Hospital}2_{ij}+\beta_{3}\mathrm{Hospital}1_{ij}+\beta_{4}\mathrm{DoctorN}F_{ij}+\\ \beta_{5}\mathrm{DoctorN}N_{ij}+\beta_{6}\mathrm{Appoint}3\mathrm{day}s_{ij}+\beta_{7}\mathrm{Appoint}7\mathrm{day}s_{ij}+\\ \beta_{8}\mathrm{Telephon}e_{ij}+\beta_{9}\mathrm{Imagetex}t_{ij}+\beta_{10}\mathrm{NonMeInsuranc}e_{ij}+\\ \gamma_{1}\cos t_{ij}\mathrm{Femal}e_{i}+\gamma_{2}\mathrm{Hospital}2_{ij}\mathrm{Femal}e_{i}+\gamma_{3}\mathrm{Hospital}1_{ij}\\ \mathrm{Femal}e_{i}+\gamma_{4}\;\mathrm{DoctorN}F_{ij}\mathrm{Femal}e_{i}+\gamma_{5}\mathrm{DoctorN}N_{ij}\mathrm{Femal}e_{i}+\\ \gamma_{6}\mathrm{Appoint}3\mathrm{day}s_{ij}\mathrm{Femal}e_{i}+\gamma_{7}\mathrm{Appoint}7\mathrm{day}s_{ij}\mathrm{Femal}e_{i}+\\ \gamma_{8}\mathrm{Telephon}e_{ij}\mathrm{Femal}e_{i}+\gamma_{9}\mathrm{Imagetex}t_{ij}\mathrm{Femal}e_{i}+\\ \gamma_{10}\mathrm{NonMeInsuranc}e_{ij}\mathrm{Femal}e_{i} \end{array}$$

Similarly, the effects of other individual characteristics can be modeled by including relevant interaction terms. All interaction terms are included as fixed variables.

## 4 Results

### 4.1 Descriptive statistical results

337 valid questionnaires were included in the analysis after data cleansing by eliminating respondents with a response time less than 3 min, choosing the same option for all DCE tasks and failed the validity check. The number of male respondents (47.2%) was slightly lower than the number of female respondents (52.8%). More than half of the respondents had a bachelor’s degree and a higher degree of education (56.1%), and almost all had medical insurance (98.2%). Most respondents lived in towns and cities (88.5%) and were employed full-time (80.5%). In addition, 46.3% of online respondents said they had no experience with online health consultations. The results of each demographic variable used in the interaction model are presented in Table 2, and more detailed information is presented in the Supplementary material.

**Table 2:** Characteristics of respondents (*N* = 337).

Characteristic	Frequency (Percentage)	Variable
Gender		
Male	159 (47.2%)	Reference
Female	178 (52.8%)	Female
Age		
18–29	64 (19.0%)	Reference
30–39	109 (32.3%)	Age 30–39
40–49	101 (30.0%)	Age 40–49
≥50	63 (18.7%)	Age ≥ 50
Education		
Below Bachelor’s degree	148 (43.9%)	Reference
Bachelor’s degree and above	189 (56.1%)	Education ≥ Bachelor’s degree
Monthly income(CNY)		
≤ 9,000	213 (63.2%)	Reference
> 9,000	124 (36.8%)	Income >9,000
Internet healthcare experience		
Yes	181 (53.7%)	Reference
No	156 (46.3%)	No experience
Chronic diseases		
Yes	123 (36.5%)	Chronic diseases
No	214 (63.5%)	Reference

### 4.2 Main effect analysis

**Figure 3:**
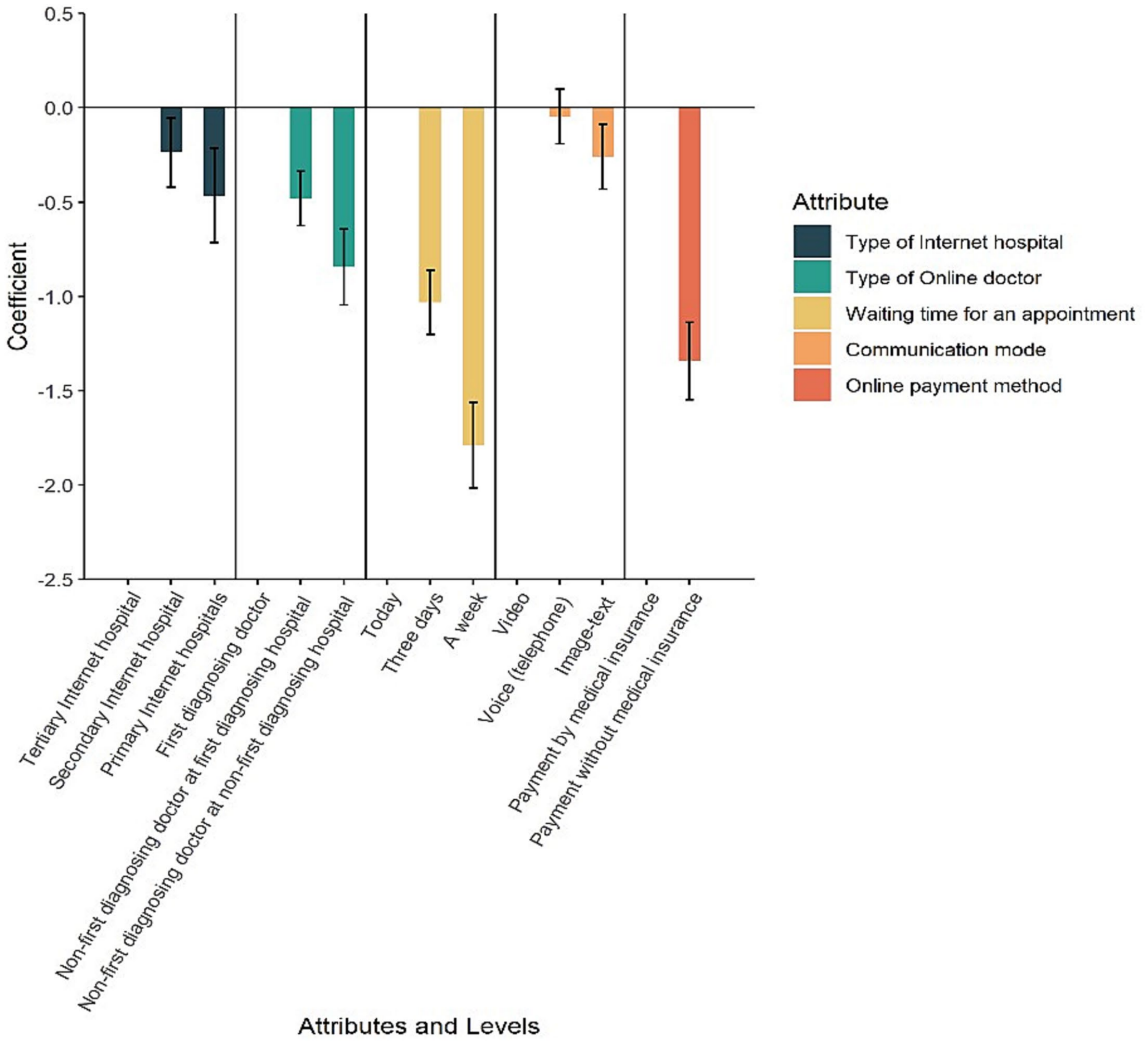
Preference weight. This figure clearly shows the effects of appointment attributes on appointment choice. Attributes with lower confidence levels tend to have stronger patient preferences. For example, respondents most preferred tertiary Internet hospitals over primary (community) Internet hospitals, followed by secondary Internet hospitals. The shorter the waiting time, the more likely respondents are to choose online follow-up visits.

A mixed logit model was estimated in Stata 17.0 with 1,000 Halton draws. The reference levels of the categorized attributes were set respectively: tertiary Internet hospital, the patient’s own first diagnosing doctor, today, video, and payment by medical insurance. The estimated coefficients can be interpreted as holding other attribute levels constant at the reference level, the average change in the respondent’s perceived utility of choice for the corresponding attribute level relative to the reference level for that attribute. Since WTP in the mixed logit model was calculated as the ratio of a non-monetary attribute level coefficient to a monetary attribute coefficient, treating cost as a random variable can result in a highly skewed distribution of WTP (50). Based on the random utility theory, the total utility of the online medical appointment option in our study can be decomposed into two parts: fixed utility and random utility. Consequently, the cost variable was specified as a fixed variable. It was assumed that the other attribute level variables were random variables following a normal distribution. We successively decide which parameters are regarded as random coefficients by testing the statistical significance of the standard deviation. Variables that did not align with the assumption of random variables for standard deviation significance (*p* < 0.05) were systematically excluded one by one as fixed effect variables. Table 3 and Figure 3 present the estimation results of the main effects model.

**Table 3:** Results of the mixed logit model for main effects.

Variables	Coefficient	Standard error	*p* value	[95% confidence interval]	
Cost	−0.030	0.003	< 0.001	−0.036	−0.024
Secondary internet hospital	−0.237	0.094	0.011	−0.421	−0.054
Primary (Community) internet hospital	−0.466	0.128	< 0.001	−0.716	−0.216
Non-first diagnosing doctor at first diagnosing hospital	−0.481	0.074	< 0.001	−0.626	−0.336
Non-first diagnosing doctor at non-first diagnosing hospital	−0.845	0.102	< 0.001	−1.045	−0.645
3 days	−1.033	0.086	< 0.001	−1.201	−0.864
A week	−1.790	0.116	< 0.001	−2.016	−1.563
Voice (Telephone) consultation	−0.047	0.074	0.524	−0.193	−0.098
Image-text consultation	−0.260	0.088	0.003	−0.432	−0.088
Payment without medical insurance	−1.342	0.104	< 0.001	−1.547	−1.138
Opt out	−6.637	0.353	< 0.001	−7.329	−5.956

#### 4.2.1 The effect of appointment attributes on appointment choice

According to Table 3, except for the *p*-value of “telephone,” which was greater than 0.05, the *p*-values of all other attribute levels were less than 0.05 or even 0.01. Respondents most preferred tertiary Internet hospitals over primary (community) Internet hospitals (coefficient = −0.466, *p* < 0.001) followed by secondary Internet hospitals (coefficient = −0.237, *p* < 0.05). Regarding the type of online doctor, respondents showed a strong preference for scheduling online outpatient follow-up appointments with their own first diagnosing doctor, rather than non-first diagnosing doctors at their first diagnosing hospitals (coefficient = −0.481, *p* < 0.001), and non-first diagnosing hospitals (coefficient = −0.845, *p* < 0.001). Respondents valued waiting 0 days (today) or 3 days for an appointment much more than waiting 7 days for an appointment (coefficient = −1.033, *p* < 0.001; coefficient = −1.790, *p* < 0.001). It is notable that respondents seemed to be less concerned about voice (phone) consultation (coefficient = −0.047, *p* = 0.524), and they preferred video consultation to image-text consultation (coefficient = −0.260, *p* < 0.01). Finally, it is clear that respondents preferred payment by medical insurance to payment without medical insurance (coefficient = −1.342, *p* < 0.001).

#### 4.2.2 Relative importance of attributes

**Figure 4:**
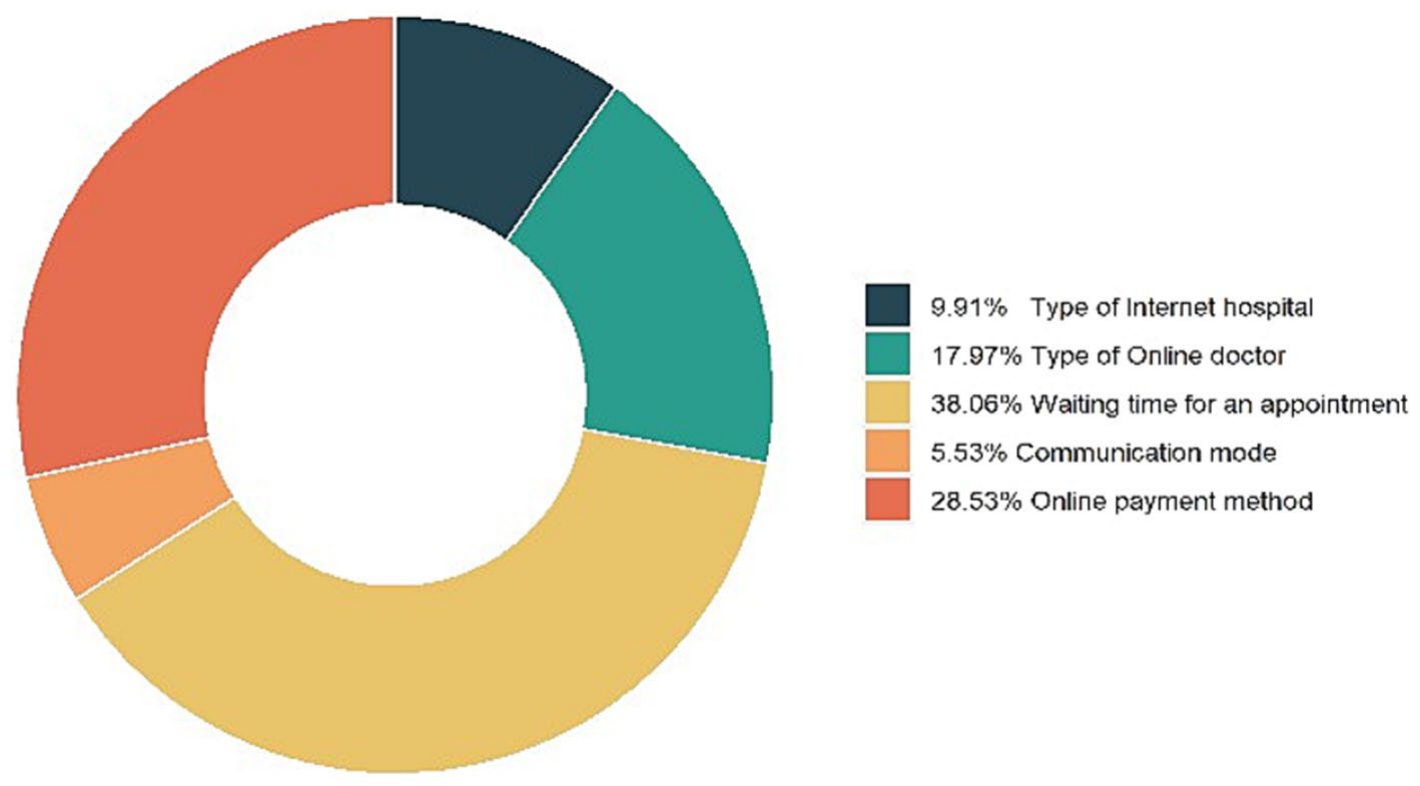
Relative importance of attributes. This figure shows the relative importance of attributes in the choice of online outpatient follow-up clinic. The most valued attribute by respondents is the waiting time for an appointment, followed by online payment method, type of online doctor, type of Internet hospital, and communication mode.

The relative importance of each attribute in the DCE indicates the relative weight of its influence on respondents’ choice preferences (36, 51). The results of the relative importance of categorical attributes other than “cost” are shown in Figure 4. In the choice of online outpatient follow-up clinics, the most valued attribute by respondents is the waiting time for an appointment, followed by online payment method, type of online doctor, type of Internet hospital, and communication mode.

#### 4.2.3 Willingness to pay

WTP is the maximum amount of money an individual is willing to spend to obtain a good or improve a service. The ratio of the coefficients of each attribute level indicates its marginal rate of substitution, so the evaluation of the monetary value of each attribute can be given by the ratio of the coefficients of each attribute level to the cost coefficient. The formula is calculated as in Equation 4:


(4) $$WTP=-\frac{\beta_{\mathrm{attributelevel}}}{\beta_{\mathrm{cost}}}$$

**Figure 5:**
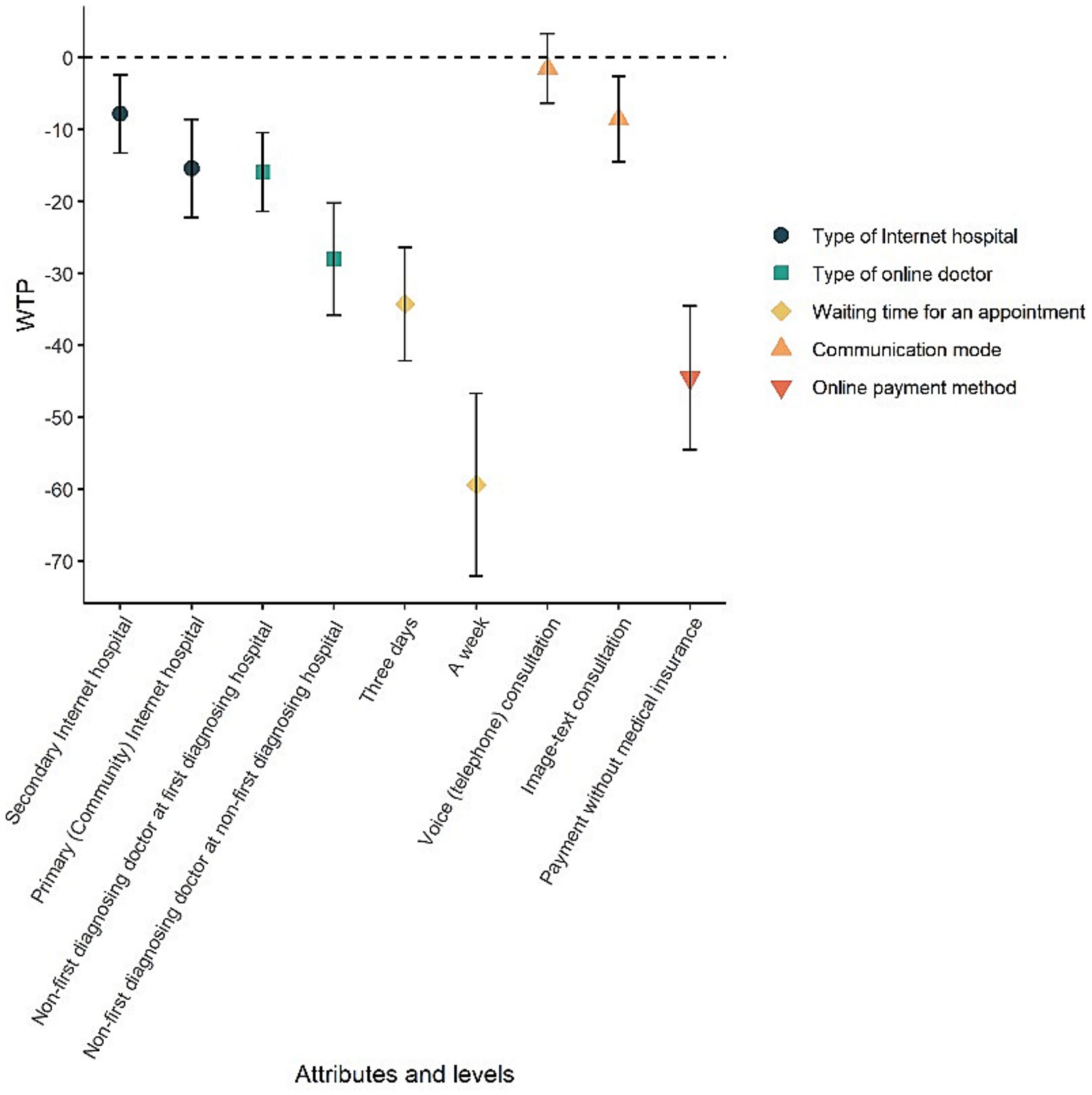
Willingness to pay. A positive WTP indicates a willingness to pay to ensure a change in the reference level, while a negative WTP means a willingness to pay to avoid a change in the reference level. This figure illustrates that respondents are willing to pay varying amounts of additional CNY to select their preferred attributes for online follow-up appointments.

A positive WTP indicates a willingness to pay to ensure a change in the reference level, while a negative WTP means to avoid a change in the reference level (51). Based on the main effects model estimation results in Table 4, the WTP results are presented in Table 4 and Figure 5. We converted the amounts in Chinese yuan in the questionnaire into US dollar based on the average exchange rate of 7.0467 in 2023, making the results of our experiment more instructive. In general, respondents were willing to pay approximately 1.11dollars and 2.18 dollars to avoid an online outpatient follow-up clinic at secondary Internet hospitals and primary (community) Internet hospitals, respectively. For the type of online doctor, respondents were willing to pay approximately 2.25 dollars to avoid a different doctor at their first diagnosing hospitals, and 3.95 dollars to avoid non-first diagnosing hospitals. Respondents were willing to pay approximately 4.82 dollars to avoid waiting 3 days and 8.36 dollars to avoid waiting a week. In addition, respondents were willing to pay an additional approximately 1.21 dollars to get a video consultation. And they were willing to pay an additional approximately 6.27 dollars to use Medicare payments.

**Table 4:** WTP estimation results.

Attribute and level	WTP(CNY) [95% confidence interval]
Type of internet hospital	
Tertiary internet hospital	Reference
Secondary internet hospital	−7.868 [−13.301, −2.435]
Primary (Community) internet hospital	−15.464 [−22.283, −8.644]
Type of online doctor	
First diagnosing doctor	Reference
Non-first diagnosing doctor at first diagnosing hospital	−15.951 [−21.435, −10.467]
Non-first diagnosing doctor at non-first diagnosing hospital	−28.025 [−35.786, −20.265]
Waiting time for an appointment	
Today	Reference
3 days	−34.250 [−42.142, −26.357]
A week	−59.359 [−72.061, −46.657]
Communication mode	
Video consultation	Reference
Voice (Telephone) consultation	−1.569 [−6.408, 3.270]
Image-text consultation	−8.616 [−14.560, −2.671]
Online payment method	
Payment by medical insurance	Reference
Payment without medical insurance	−44.527 [−54.526, −34.528]

### 4.3 Interaction model analysis

The effects of 6 categorical variables (gender, age, education, income, Internet healthcare experience, and chronic disease status) and 4 continuous variables (RA, HTSE, EHEAL, and OPC) on preferences across attribute levels were highlighted. The interaction terms were included as fixed-effect variables in a mixed logit model, again with 1,000 Halton draws. Interaction model 1 in Table 5 recorded the final reduced interaction model results (only the significant interaction terms with *p* < 0.05 were retained). As a robustness test of Interaction model, Interaction model 2 estimated a new mixed logit model including all main effects and significant (p < 0.05) interaction terms from Interaction model 1.

**Table 5:** Results of the mixed logit model for interaction effects.

Variables	Interaction model1		Interaction model2	
	Coefficient	*p* value	Coefficient	*p* value
Cost	−0.030	0.259	0.003	0.786
Secondary internet hospital	−0.067	0.945	−0.253*	0.025
Non-first diagnosing doctor at first diagnosing hospital	−1.048	0.203	−0.608	0.230
3 days	−1.915*	0.026	−1.211**	<0.001
Voice (telephone) consultation	−0.330	0.694	−0.122	0.134
Cost*Chronic diseases	0.016**	0.003	0.010*	0.013
Cost*HTSE	−0.009*	0.011	−0.007**	0.002
Secondary Internet hospital *Age 40–49	−0.751*	0.015	−0.335*	0.035
Secondary Internet hospital *Income >9,000	0.438*	0.033	0.323*	0.033
Non-first diagnosing doctor at first diagnosing hospital*Chronic diseases	−0.328*	0.042	−0.305*	0.036
Non-first diagnosing doctor at first diagnosing hospital*HTSE	−0.257*	0.020	−0.197*	0.043
Non-first diagnosing doctor at first diagnosing hospital*EHEAL	0.516**	0.007	0.337*	0.044
Non-first diagnosing doctor at non-first diagnosing hospital*Female	−0.436*	0.038	−0.227	0.204
Non-first diagnosing doctor at non-first diagnosing hospital* Chronic diseases	−0.580**	0.007	−0.448*	0.024
Non-first diagnosing doctor at non-first diagnosing hospital*EHEAL	0.577*	0.024	0.112	0.506
3 days*Female	0.416*	0.011	0.307*	0.030
Voice (Telephone) consultation *Age ≥ 50	0.691*	0.025	0.405*	0.034
Image-text consultation *Age 30–39	0.758**	0.008	0.137	0.538
Image-text consultation *Age 40–49	0.679*	0.031	0.192	0.395
Image-text consultation *Age ≥ 50	0.731*	0.044	0.279	0.297
Primary (Community) Internet hospital	−1.901	0.126	−0.442**	0.001
Non-first diagnosing doctor at non-first diagnosing hospital	−2.328*	0.033	−0.920	0.175
A week	−0.825	0.470	−1.817**	<0.001
Image-text consultation	−1.490	0.133	−0.415*	0.021
Payment without medical insurance	−1.519	0.184	−1.327**	<0.001
Opt out	−6.837**	<0.001	−6.540**	<0.001

The results of interaction models are shown in Table 5. Some of the interactions were statistically significant (*p* < 0.05), suggesting that individual characteristics had an effect on patient preferences. The results of interaction model 2 were used as the final interpretation of the heterogeneity analysis. For example, the coefficient for “Cost*Chronic diseases” was 0.01 (*p* < 0.05), which could be interpreted to mean that chronic patients were relatively more likely to accept high costs. The coefficient for “Cost*HTSE” was −0.007 (*p* < 0.01), because those with higher HTSE were less willing to spend more money. In terms of the type of Internet hospital, respondents aged 40–49 were less inclined to choose secondary Internet hospitals (compared to tertiary Internet hospitals) than respondents aged 18–29 (coefficient = −0.335, *p* < 0.05). While respondents with higher monthly income (> 1267.6 dollars) were more likely to accept appointments at secondary Internet hospitals (coefficient = −0.323, *p* < 0.05). Respondents with chronic diseases had a strong preference not to make online outpatient follow-up appointments with doctors other than their own first diagnosing doctors (coefficient = −0.305, *p* < 0.05; coefficient = −0.448, *p* < 0.05). Individuals with higher HTSE were also disinclined to choose a doctor other than the one who first diagnosed them in the hospital where they were first diagnosed (coefficient = −0.197, *p* < 0.05). However, those with higher EHEAL were more accepting of choosing a doctor other than their own for follow-up appointments (coefficient = 0.337, *p* < 0.05). In addition, Females showed greater tolerance for waiting 3 days for an appointment than males (coefficient = −0.307, *p* < 0.05). Respondents aged 50 and over were more likely to accept telephone consultations than those aged 18–29 (coefficient = −0.405, *p* < 0.05).

## 5 Discussion

This study evaluated the preferences of respondents in China regarding the attributes of online outpatient follow-up clinics. The results revealed that all six attributes influenced the choice of respondents, of which shorter waiting time and payment by medical insurance are the most crucial factors. Respondents expressed a preference for an online outpatient follow-up clinic characterized by low cost, offered by a public tertiary Internet hospital, attended by doctors who first diagnosed them, shorter waiting time, video consultations, and paying costs by medical insurance. Age, chronic disease, HTSE and EHEAL influenced the strength of preference for the attributes.

Previous studies have identified two especially critical factors that patients consider when scheduling outpatient appointments: quality and speed. Patients may reduce their waiting time for appointments with a non-regular doctor, but seeing an unfamiliar doctor can disrupt continuity of care, potentially diminishing its quality (48). In our study, the relative importance of waiting time was highest, followed by online payment methods and the continuity of doctors. This meant that Chinese respondents valued waiting time more than continuity of online doctors. It was consistent with the results of a labeled DCE conducted by Chudner et al. (52), which found that Israeli patients placed less importance on the relationship with the physician than the time to the next available appointment and quality of consultation. Online payment method was the second most important attribute following waiting time, with Chinese respondents placing great importance on Medical payment. This also supported the findings of Zhang et al. (51), their results showed that Chinese respondents valued the type of provider the most, followed by reimbursement rates. Collectively, respondents also considered the type of Internet hospital to be an important factor, with tertiary Internet hospitals being the most preferred. Because tertiary hospitals typically possess more advanced medical facilities, a wider range of specialties, highly skilled medical staff and long-established reputation. This finding also aligns with the study by Zhu et al. (53), which found that Chinese outpatients most preferred tertiary hospitals in the non-telehealth context. The consistent findings of the two studies suggest that, irrespective of whether the healthcare service is delivered in-person or online, patients generally attach great importance to the hospital’s level.

Communication mode influenced the choice of online outpatient follow-up clinics, although its significance was relatively lower than other attributes. This study revealed that respondents had a strong preference for video consultations over image-text consultations, and the coefficient for voice (phone) consultations did not show statistical significance in the main effects model. This may indicate that respondents value importance on the efficiency and quality of communication with their care providers. This reinforces the factor that cost is not an issue compared to what patients prefer. Similarly, numerous studies have demonstrated the importance of doctor-patient communication and healthcare quality (14, 52, 54, 55). Several studies have found that patients seem to prefer video consultations over telephone consultations (22, 56, 57). A review found that videoconferencing was more beneficial than telephone in healthcare delivery, with higher diagnostic accuracy (57).

Patient priorities can be used by healthcare providers to improve the sensitivity of public Internet hospitals to satisfy patient demand, improve the delivery of medical services and set priorities for ensuring service quality. Optimizing the scheduling of online doctors, adjusting opening hours and the quantity of online appointments, and striving to reduce patients’ waiting time for online care compared to offline care can maximize the impact of telehealth on enhancing patient accessibility and convenience. The government should establish clear specifications for Internet medical reimbursement to expedite the implementation of online Medicare payment across all levels of existing public Internet hospitals. Recognizing the significance of continuity for follow-up care, doctors can cultivate enduring relationships with their patients in need. Empirical results indicated that, in comparison to tertiary Internet hospitals, patients exhibit the least preference for online access to primary (community) Internet hospitals and for online consultations through image-text consultations (as opposed to video consultations). This aligns with findings from Gong et al. (58), who reported that 78.6% of patients preferred tertiary hospitals due to perceived higher quality and expertise. Similarly, Ding et al. (9) found that 68.3% of patients favored video consultations over image-text consultations, citing the lack of real-time interaction as a major drawback. In practice, it is true that China’s primary healthcare institutions face challenges in terms of basic diagnostic facilities and hospital information system. Hence, it is imperative to redesign the respective focus and improve operational mode (59–61) of Internet hospitals at all levels to enhance the delivery of telehealth services, doctor-patient communication, and overall quality of care.

This study also has several limitations. Firstly, patients’ decisions and preferences regarding telehealth are complex and can be influenced directly or indirectly by factors that have not been taken into account in actual healthcare settings. Secondly, the respondents are recruited online, implying a level of Internet proficiency. These online respondents are more educated, fewer are over 50 and most live in urban areas. Future studies should pay special attention to the preferences for telehealth among older adult and rural individuals. Thirdly, in our survey, we adopted monetary incentives to attract a large number of participants to fill in the questionnaire. Whether these incentives would have an impact on the patients’ preferences for online outpatient follow-up clinics is worthy of further exploration. Finally, this study focuses on patients’ preferences for online outpatient follow-up clinics provided by public Internet hospitals. Therefore, some of the attributes and attribute levels developed may not accurately represent the service characteristics of other types of Internet hospitals, especially those established by enterprises.

## 6 Conclusion

This study used the DCE method to elicit respondents’ preferences for online outpatient follow-up clinics provided by public Internet hospitals in China. The empirical results showed that all six attributes (cost, type of Internet hospital, type of online doctor, waiting time for an appointment, communication mode, and online payment method) had a statistically significant impact on the choice of online follow-up appointments. Among these attributes, waiting time for an appointment was the most crucial for respondents, followed by online payment method, type of online doctor, type of Internet hospital, and communication mode. The results of WTP suggested that when other attributes are controlled at the baseline level, respondents were willing to pay the most to avoid a seven-day appointment delay, and they are willing to pay the least for a video consultation. Furthermore, the study identified sources of heterogeneity in respondents’ preferences, including the presence of age, chronic diseases, HTSE and EHEAL, which are significant at 0.05 level in our interaction model. This study provides preliminary indications that Chinese patients place a high priority on the ability to access healthcare services quickly and reduce the cost of care when seeking healthcare online, as they are willing to pay the most for reducing waiting time and then using medical insurance for payment.

## Data Availability

The raw data supporting the conclusions of this article will be made available by the authors, without undue reservation.

## Footnotes

^1^ www.wjx.cn
