# Supplementary material for: A determination of patient preferences for China online outpatient follow-up clinics by using discrete choice experiment: an exploratory study
Source: Front Public Health. 2025 Mar 24;13:1508369. doi: 10.3389/fpubh.2025.1508369 (PMC11973272; doi:10.3389/fpubh.2025.1508369)
Supplement: Supplementary file 1 [file Table_1.docx]

Supplementary MaterialSupplementary Figures and Tables

## Supplementary Tables

Results of respondent demographics

**Table A1.** Characteristics of respondents (N=337).

| **Characteristics** | **Frequency (Percentage)** | |
| --- | --- | --- |
| Gender |  |  |
| male | 159 (47.2%) |  |
| female | 178 (52.8%) |  |
| Age |  |  |
| 18–29 | 64 (19.0%) |  |
| 30–39 | 109 (32.3%) |  |
| 40–49 | 101 (30.0%) |  |
| ≥50 | 63 (18.7%) |  |
| Education |  |  |
| Junior high school and below | 18 (5.3%) |  |
| High school | 52 (15.4%) |  |
| University colleges (including those in study) | 78 (23.1%) |  |
| Bachelor’s degree (including those in study) | 168 (49.9%) |  |
| Master's degree students (including those in study) | 20 (5.9%) |  |
| PhD (including those in study) | 1 (0.3%) |  |
| Monthly income(CNY） |  |  |
| ≤ 3,000 | 19 (5.6%) |  |
| 3001–6000 | 85 (25.2%) |  |
| 6001–9,000 | 109 (32.3%) |  |
| 9001–12,000 | 64 (19.0%) |  |
| 12,001–15,000 | 31 (9.2%) |  |
| ＞ 15,000 | 29 (8.6%) |  |
| Internet healthcare experience |  |  |
| Yes | 181 (53.7%) |  |
| No | 156 (46.3%) |  |
| Chronic diseases |  |  |
| Yes | 123 (36.5%) |  |
| No | 214 (63.5%) |  |
| Occupation |  |  |
| Self-employed | 20 (5.9%) |  |
| Full-time employment | 271 (80.4%) |  |
| Part-time employment | 11 (3.3%) |  |
| Retirement | 33 (9.8%) |  |
| Other | 2 (0.6%) |  |
| Residence |  |  |
| Town/city | 298 (88.4%) |  |
| Rural areas | 39 (11.6%) |  |
| Medical insurance |  |  |
| Yes | 331 (98.2%) |  |
| No | 6 (1.8%) |  |
| Perceived health status |  |  |
| Very good | 34 (10.1%) |  |
| Good | 174 (51.6%) |  |
| General | 103 (30.6%) |  |
| Poor | 25 (7.4%) |  |
| Very poor | 1 (0.3%) |  |
| Number of hospital visits in the past year |  |  |
| ≤ 3 | 213 (63.2%) |  |
| 4–6 | 97 (28.9%) |  |
| 7–9 | 20 (5.9%) |  |
| ≥ 10 | 7 (2.1%) |  |
| Knowledge of public Internet hospitals |  |  |
| Very little | 17 (5.0%) |  |
| Less | 36 (10.7%) |  |
| General | 132 (39.2%) |  |
| More | 129 (38.3%) |  |
| A lot | 23 (6.8%) |  |
| Trust in public Internet hospitals |  |  |
| Very little | 4 (1.2%) |  |
| Less | 11 (3.3%) |  |
| General | 72 (21.4%) |  |
| More | 203 (60.2%) |  |
| A lot | 47 (13.9%) |  |

Results of the Risk Attitude (RA) Scale, Healthcare Technology Self-Efficacy (HTSE) Scale, E-Health Literacy (EHEAL) Scale, and Online Privacy Concerns (OPC) Scale

**Table B1.** Risk-Taking (DOSPERT) scale- Health/Safety (N=337)

| **Item** | **Percentage** |
| --- | --- |
| Q1: Drinking heavily at a social function. |  |
| Extremely Unlikely | 157(46.6%） |
| Moderately Unlikely | 92(27.3%） |
| Somewhat Unlikely | 37(11.0%） |
| Not Sure | 10(3.0%） |
| Extremely Likely | 26(7.7%） |
| Moderately Likely | 11(3.3%） |
| Somewhat Likely | 4(1.2%） |
| Q2: Engaging in unprotected sex. |  |
| Extremely Unlikely | 151(44.8%） |
| Moderately Unlikely | 69(20.5%） |
| Somewhat Unlikely | 39(11.6%） |
| Not Sure | 33(9.8%） |
| Extremely Likely | 27(8.0%） |
| Moderately Likely | 15(4.5%） |
| Somewhat Likely | 3(0.9%） |
| Q3: Driving a car without wearing a seat belt. |  |
| Extremely Unlikely | 210(62.3%） |
| Moderately Unlikely | 71(21.1%） |
| Somewhat Unlikely | 27(8.0%） |
| Not Sure | 6(1.8%） |
| Extremely Likely | 15(4.5%） |
| Moderately Likely | 4(1.2%） |
| Somewhat Likely | 4(1.2%） |
| Q4: Riding a motorcycle without a helmet. |  |
| Extremely Unlikely | 128(38.0%） |
| Moderately Unlikely | 94(27.9%） |
| Somewhat Unlikely | 46(13.6%） |
| Not Sure | 27(8.0%） |
| Extremely Likely | 26(7.7%） |
| Moderately Likely | 13(3.9%） |
| Somewhat Likely | 3(0.9%） |
| Q5: Sunbathing without sunscreen. |  |
| Extremely Unlikely | 56(16.6%） |
| Moderately Unlikely | 67(19.9%） |
| Somewhat Unlikely | 49(14.5%） |
| Not Sure | 44(13.1%） |
| Extremely Likely | 66(19.6%） |
| Moderately Likely | 38(11.3%） |
| Somewhat Likely | 17(5.0%） |
| Q6: Walking home alone at night in an unsafe area of town. |  |
| Extremely Unlikely | 72(21.4%） |
| Moderately Unlikely | 87(25.8%） |
| Somewhat Unlikely | 54(16.0%） |
| Not Sure | 52(15.4%） |
| Extremely Likely | 53(15.7%） |
| Moderately Likely | 11(3.3%） |
| Somewhat Likely | 8(2.4%） |

**Table B2.** Healthcare Technology Self-Efficacy (HTSE) Scale (N=337).

| **Item** | **Percentage** |
| --- | --- |
| Q1: It is easy for me to use internet health services. |  |
| Strongly Disagree | 2(0.6%） |
| Very Disagree | 2(0.6%） |
| Somewhat Disagree | 9(2.7%） |
| General | 39(11.6%） |
| Somewhat Agree | 109(32.3%） |
| Very Agree | 141(41.8%） |
| Strongly Agree | 35(10.4%） |
| Q2: I feel uncomfortable to use internet health services. |  |
| Strongly Disagree | 37(11.0%） |
| Very Disagree | 104(30.9%） |
| Somewhat Disagree | 126(37.4%） |
| General | 50(14.8%） |
| Somewhat Agree | 11(3.3%） |
| Very Agree | 8(2.4%） |
| Strongly Agree | 1(0.3%） |
| Q3: I am very confident in my abilities to use internet health services. |  |
| Strongly Disagree | 3(0.9%） |
| Very Disagree | 5(1.5%） |
| Somewhat Disagree | 10(3.0%） |
| General | 39(11.6%） |
| Somewhat Agree | 103(30.6%） |
| Very Agree | 114(33.8%） |
| Strongly Agree | 63(18.7%） |
| Q4: I would be able to use internet health services without much effort. |  |
| Strongly Disagree | 1(0.3%） |
| Very Disagree | 10(3.0%） |
| Somewhat Disagree | 15(4.5%） |
| General | 46(13.6%） |
| Somewhat Agree | 118(35.0%） |
| Very Agree | 106(31.5%） |
| Strongly Agree | 41(12.2%） |

**Table B3.** EHealth Literacy Scale (N=337).

| **Item** | **Percentage** |
| --- | --- |
| Q1: I know how to find helpful health resources on the Internet |  |
| Strongly Disagree | 2(0.6%） |
| Disagree | 12(3.6%） |
| Undecided | 58(17.2%） |
| agree | 213(63.2%） |
| Strongly agree | 52(15.4%） |
| Q2: I know how to use the Internet to answer my health questions |  |
| Strongly Disagree | 2(0.6%） |
| Disagree | 18(5.3%） |
| Undecided | 51(15.1%） |
| agree | 173(51.3%） |
| Strongly agree | 93(27.6%） |
| Q3: I know what health resources are available on the Internet |  |
| Strongly Disagree | 4(1.2%） |
| Disagree | 13(3.9%） |
| Undecided | 58(17.2%） |
| agree | 164(48.7%） |
| Strongly agree | 98(29.1%） |
| Q4: I know where to find helpful health resources on the Internet |  |
| Strongly Disagree | 1(0.3%） |
| Disagree | 11(3.3%） |
| Undecided | 55(16.3%） |
| agree | 173(51.3%） |
| Strongly agree | 97(28.8%） |
| Q5: I know how to use the health information I find on the Internet to help me |  |
| Strongly Disagree | 0(0%） |
| Disagree | 3(0.9%） |
| Undecided | 46(13.6%） |
| agree | 176(52.2%） |
| Strongly agree | 112(33.2%） |
| Q6: I have the skills I need to evaluate the health resources I find on the Internet |  |
| Strongly Disagree | 5(1.5%） |
| Disagree | 23(6.8%） |
| Undecided | 93(27.6%） |
| agree | 145(43.0%） |
| Strongly agree | 71(21.1%） |
| Q7: I can tell high quality from low quality health resources on the Internet |  |
| Strongly Disagree | 9(2.7%） |
| Disagree | 41(12.2%） |
| Undecided | 100(29.7%） |
| agree | 117(34.7%） |
| Strongly agree | 70(20.8%） |
| Q8: I feel confident in using information from the Internet to make health decisions |  |
| Strongly Disagree | 4(1.2%） |
| Disagree | 31(9.2%） |
| Undecided | 75(22.3%） |
| agree | 148(43.9%） |
| Strongly agree | 79(23.4%） |

**Table B4.** Online Privacy Concerns Scale (N=337).

| **Item** | **Percentage** |
| --- | --- |
| Q1: Are you concerned that you are asked for too much personal information when you register or make online purchases? |  |
| Fully concerned | 10(3.0%） |
| Rather concerned | 46(13.6%） |
| Neither concerned nor not concerned | 50(14.8%） |
| Rather not concerned | 171(50.7%） |
| Not at all concerned | 60(17.8%） |
| Q2: Are you concerned that an email you send may be read by someone else besides the person you sent it to? |  |
| Fully concerned | 24(7.1%） |
| Rather concerned | 50(14.8%） |
| Neither concerned nor not concerned | 69(20.5%） |
| Rather not concerned | 107(31.8%） |
| Not at all concerned | 87(25.8%） |
| Q3: Are you concerned that if you use your credit card to buy something on the internet your card will be mischarged? |  |
| Fully concerned | 27(8.0%） |
| Rather concerned | 50(14.8%） |
| Neither concerned nor not concerned | 76(22.6%） |
| Rather not concerned | 93(27.6%） |
| Not at all concerned | 91(27.0%） |
| Q4: Are you concerned who might access your medical records electronically? |  |
| Fully concerned | 23(6.8%） |
| Rather concerned | 53(15.7%） |
| Neither concerned nor not concerned | 62(18.4%） |
| Rather not concerned | 136(40.4%） |
| Not at all concerned | 63(18.7%） |
